# Measuring Parallelism to the Ground in Bipedal Stance Phase: Mechanical Versus Kinematic Alignment in Total Knee Arthroplasty

**DOI:** 10.7759/cureus.55173

**Published:** 2024-02-28

**Authors:** Malaak Hamzeh, Kaelyn Gwynne, Brian J Panish, Bradley Gelfand, Evan Argintar

**Affiliations:** 1 Orthopaedic Surgery, Georgetown University School of Medicine, Washington, DC, USA; 2 Orthopaedic Surgery, MedStar Georgetown University Hospital, Washington, DC, USA; 3 Orthopaedic Surgery, MedStar Washington Hospital Center, Washington, DC, USA

**Keywords:** weight bearing, bipedal stance phase, tibial-ankle axis, kinematically aligned total knee arthroplasty, total knee replacement (tkr), parallelism, kinematic axis, joint line, kinematic alignment, knee arthroplasty

## Abstract

Introduction

The goal of total knee arthroplasty is to replace diseased cartilage and bone with an artificial implant to improve the patient’s quality of life. The knee has historically been reconstructed to the patient’s mechanical axis (MA). However, kinematically aligned techniques have been increasingly used. Kinematic alignment requires less soft-tissue resection and aligns the knee with what is anatomically natural to the patient, while there is concern that kinematically aligned knees will lead to earlier failure due to potential unequal weight distribution on the implant. The purpose of this study is to compare the parallelism from the floor of the joint-line cuts using kinematic and mechanical alignment and understand if the MA is a proper estimation of the tibial-ankle axis (TA).

Methods

A retrospective study was conducted by recruiting all high tibial osteotomy and distal femoral osteotomy recipients operated on by two surgeons in two MedStar Health hospitals from 01/2013 to 07/2020 with full-length films in preparation for restorative procedures. Baseline osteoarthritis was graded using the Kellgren-Lawrence classification system with all patients presenting as Grade 0. The TA and the joint-line orientations of the MA and kinematic axis (KA) were measured on 66 legs. The average distance from parallelism to the ground was compared between the MA and the KA and between the MA and the TA using a paired t-test.

Results

KA joint-line orientation (1.705° deviation) was more parallel to the floor in the bipedal stance phase than the MA (2.316° deviation, p=0.0156). The MA (2.316° deviation) was not a proper estimation of the TA (4.278° deviation, p=0.0001).

Conclusion

By utilizing the KA technique, the restoration of the natural joint line, as well as a joint that is more parallel to the floor in the stance phase compared to the MA, is achieved. The parallelism to the ground of the KA during the bipedal stance phase suggests an even load distribution across the knee. In addition, due to its similarity to the KA and anatomical significance in weight-bearing distribution, further investigation into the hip-to-calcaneal axis as an approximation of the joint line is warranted.

## Introduction

The goal of a total knee arthroplasty (TKA) is to replace diseased cartilage and bone with an artificial implant to improve the quality of life of the patient [1]. Nonetheless, roughly 19% of primary TKA patients report significant dissatisfaction. Despite the overall success of TKA, there is an evident need for advancement in improving patient outcomes.

Historically, a mechanically aligned TKA has been the standard method of knee arthroplasty. The procedure is centered on the concept of making bone cuts based on the patient’s mechanical axis (MA). Bone alignment based on the MA combined with soft-tissue balancing can equalize the load distribution on the implant. Through making cuts perpendicular to the axis between the center of the femoral head and the center of the ankle, the belief is that the mechanical pressure on the prosthesis is evenly distributed [2]. However, the mechanically aligned knee can alter the patient’s natural anatomy, which may require significant soft-tissue releases and fail to address patient-specific differences in knee orientation [3]. This has sparked innovation of TKA techniques that are more patient-specific [4]. 

This dilemma of creating an unnatural joint-line orientation in mechanically aligned TKA led researchers to explore the concept of individualized anatomical restoration utilization of the kinematic axis (KA) [4,5]. By reconstructing the knee to its KA, the femoral-tibial axis and joint-line orientation are restored to the constitutional alignment of the patient’s pre-arthritic knee [4,6]. One main critique of the kinematically aligned TKA is that its resultant joint line is often placed in 3° varus, which opposes the long-held belief that implant survivorship is dependent upon neutral alignment [7,8]. However, outcomes of studies comparing outcomes of kinematic versus mechanical TKAs have not shown this expected early implant failure in patients with kinematically aligned implants [9]. Studies have hypothesized that the valgus pull on the knee during the bipedal stance phase may shift the alignment of the varus kinematic knee more neutrally during this gait phase, which aligns more closely with the belief that neutral alignment equalizes load distribution on the implant [8].

In a landmark study, Bellemans et al. found that 17% of asymptomatic patients had a constitutional alignment of 3° varus or more, suggesting that neutral alignment after TKA would be unnatural and alter patients’ native anatomy in a substantial proportion of the population [1]. Another study found that the joint line is often parallel to the ground in the bipedal stance phase of the native knee [10]. This study brought to attention the utility of measuring the joint line’s postoperative parallelism to the ground, considering its relationship to the distribution of load force and decrease in sheer stress [10,11]. Some studies suggested that the reconstructed joint line’s parallelism to the ground in the bipedal stance phase may even play a role in patient satisfaction, clinical outcomes, and the creation of a knee that feels more normal [3,6,12,13]. Despite the clinical and biomechanical implications of joint-line parallelism, the present literature mainly measures coronal alignment relative to the MA. Our study aims to fill the gap in the literature regarding joint-line parallelism to the ground and its relationship to the KA.

The purpose of this study is to (1) compare the parallelism of theoretical joint-line cuts with the floor using kinematic versus mechanical alignment; (2) understand how to approximate the optimal knee alignment in regard to parallelism; (3) compare the differences between the MA and KA with respect to one another; and (4) understand if the MA is a proper estimation of the tibial-ankle axis (TA).

## Materials and methods

This retrospective study was approved by the Institutional Review Board of Georgetown University School of Medicine. For the study, we recruited all high tibial osteotomy and distal femoral osteotomy recipients operated on by two surgeons in two Medstar Health hospitals from 01/2013 to 07/2020. All patients had full-length films in preparation for osteotomy in addition to other restorative procedures - meniscal transplant or large osteochondral transplant. These preoperative radiographs were used for analysis in this study. Baseline osteoarthritis was graded using the Kellgren Lawrence classification system with all patients presenting as Grade 0 [14,15]. A total of 51 patients were identified, while 14 patients were excluded due to incomplete full-length imaging and three patients were removed due to severe deformity secondary to skeletal dysplasia. Of these 34 recipients, only 66 legs were included in the study, as two had severe compartmental degenerative changes in the contralateral extremity.

We used the embedded functions of Inkscape (Free Software Foundation, Boston, MA), which is a vector graphics editor used to create vector images, primarily in Scalable Vector Graphics format, to make angular measurements. It was done on a 32-inch monitor in landscape mode. The software could detect minimum angle differences under 0.001°. Radiographs were analyzed by one researcher and measurements were repeated to ensure intra-observer reliability.

**Figure 1:**
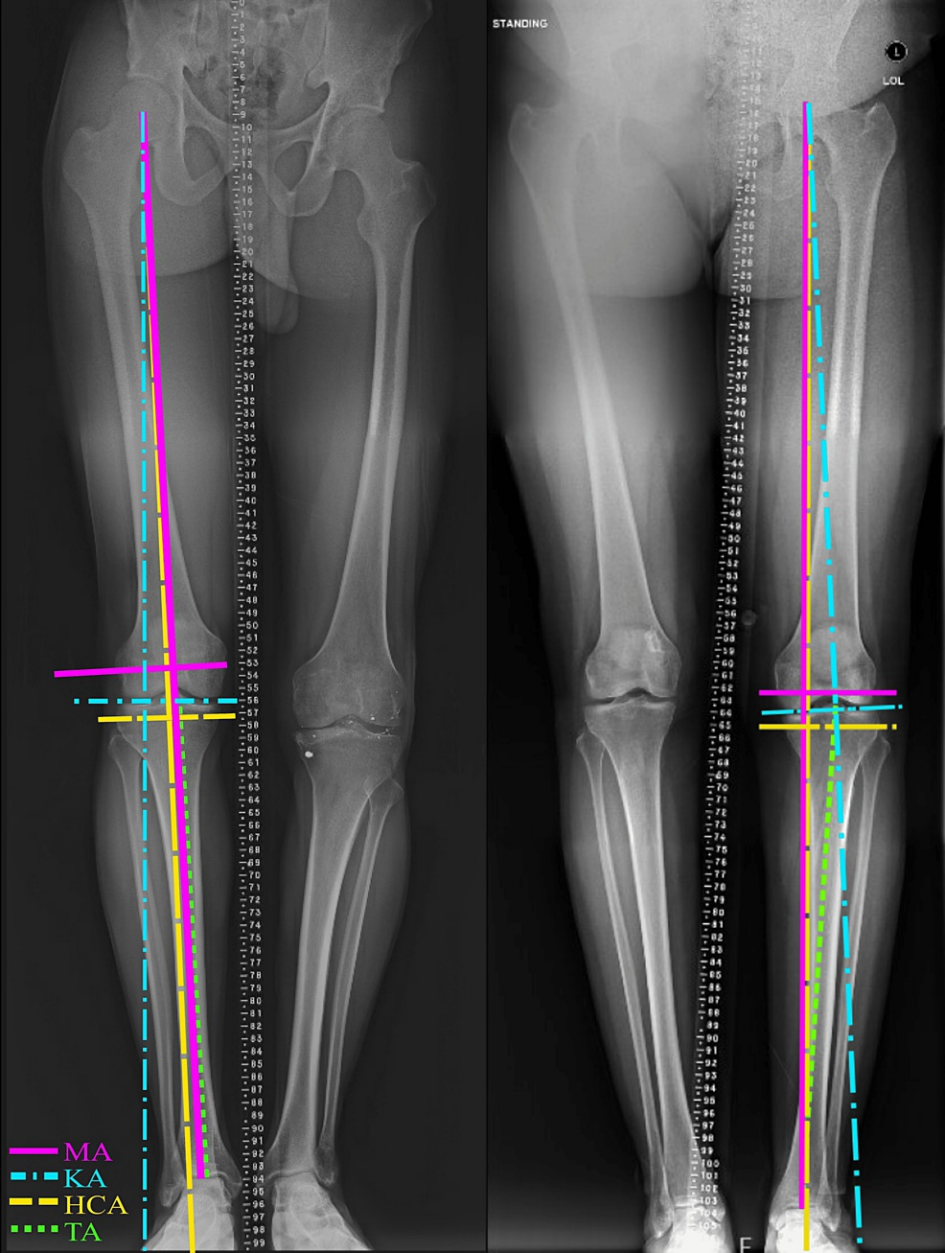
Two full bone-length standing hip-to-ankle digital radiographs of two different patients that were analyzed, depicting the mechanical, kinematic, hip-to-calcaneus, and tibial-ankle axes. MA=mechanical axis and its perpendicular joint-line; KA=kinematic axis and its perpendicular joint-line; HCA=hip-to-calcaneus axis and its perpendicular joint-line; TA=tibial-ankle axis.

To estimate the MA, a line was drawn from the center of the femoral head to the center of the talus. A line perpendicular to the MA was made at the knee joint, and that line’s parallelism to the floor was measured. To estimate the KA, a line between the lateral and medial condyle of the femur was created, and its parallelism to the floor was measured. To determine where the KA intersects the distal foot, a line perpendicular to the KA joint line was drawn from the center of the femoral head, where the MA begins, to the distal foot. The hip-to-calcaneus axis was measured by drawing a line through the center of the femoral head to the lowest point of the calcaneus. To estimate the TA, a line from the center of the intercondylar eminence and the center of the distal tibia was made (Figure 1).

The MA and KA’s parallelism to the floor was measured in degrees and converted from true values (i.e. -1.33°) to absolute values, as the absolute difference from parallel was of interest. The average distances from the parallel of the KA and the MA were calculated and then compared using a paired t-test (JMP Pro v15, SAS, Cary, North Carolina). Statistical significance was set at p <0.05. The mean, standard deviation (SD), and 95% confidence intervals (CI) were determined for each measure in each group. The same protocol was used in the comparison of the average distance from the parallel of the MA and the TA. To determine the average location of where the KA intersects the distal foot if starting at the femoral head, we calculated the average angular difference between the MA and the KA as an estimate of the angular deviation from the mechanical hip-to-ankle axis. For this comparison, the absolute angular difference between the KA and the MA was calculated and the average difference of the 66 legs was analyzed.

## Results

This study analyzed the full-length preoperative radiographs of 66 legs. The average angular difference between MA and KA was 2.957° (95% CI 2.488-3.427) in the valgus direction, often landing near the calcaneus when starting the axis at the center of the femoral head. The average angular difference between MA and TA was 2.598° (95% CI 2.075-3.121) (Table 1).

**Table 1:** Average angular difference between the mechanical axis, kinematic axis, and tibial-ankle axis SD=standard deviation; SEM=standard error of the mean; CI=confidence interval.

	Mechanical vs. kinematic axis	Mechanical vs. tibial-ankle axis
Average angular difference	2.957°	2.598°
SD	1.909	2.127
SEM	0.235	0.262
95% CI	2.488-3.427	2.075-3.121
Sample size	66	66

Of the KA, MA, and TA, the kinematic joint line was the most parallel to the floor in the bipedal stance phase, deviating from perfect parallelism by 1.705° (95% CI 1.368-2.042). The mechanical joint line deviated from perfect parallelism by 2.316° (95% CI 1.954-2.677) while a TA joint line deviated by 4.278° (95% CI 3.772-4.783) (Table 2). 

**Table 2:** Average angular difference to parallel of the kinematic, mechanical, and tibial-ankle axes SD=standard deviation; SEM=standard error of the mean; CI=confidence interval.

	Kinematic axis	Mechanical axis	Tibial-ankle axis
Average angular deviation from parallel	1.705°	2.316°	4.278°
SD	1.371	1.471	2.057
SEM	0.169	0.181	0.253
95% CI	1.368-2.042	1.954-2.677	3.772-4.783
Sample size	66	66	66

A paired t-test showed that the KA joint line was significantly more parallel to the ground than the MA (p=0.0156). The MA parallelism to the ground was significantly different from the TA parallelism to the ground (p=0.0001) (Table 3).

**Table 3:** Paired t-test comparing average angular difference to parallel in stance phase between the mechanical versus kinematic axis and the mechanical versus tibial-ankle axis CI=confidence interval. *Indicates significance.

	Kinematic vs. mechanical axis	Kinematic vs. tibial-ankle axis
Mean difference	0.610°	1.962°
95% CI	0.056-1.164	1.489-2.434
p-Value	0.0156*	0.0001*

## Discussion

Our study showed that the kinematic joint line, which restores the patient-specific constitutional joint line, is more parallel to the floor in the bipedal stance phase than the conventional mechanically aligned joint. Additionally, the secondary outcome of our study showed that the MA is not a proper estimation of the anatomic TA. Thus, we rejected our primary hypothesis stating that there was no significant difference between parallelism to the ground of the MA and KA. In addition, we rejected the secondary null hypothesis predicting that the MA was a proper estimation of the TA.

Considering that the number of TKAs performed in the United States is estimated to increase by 673% before 2030, it is paramount to improve the outcomes of this surgery in a cohort of patients who have historically been unsatisfied with clinical outcomes [16]. Postoperatively, 66% of patients indicated that their knees felt normal, 33% reported some degree of pain, 41% reported stiffness, 33% reported grinding/other noises, 33% reported swelling/tightness, 38% reported difficulty getting in and out of a car, 31% reported difficulty getting in and out of a chair, and 54% reported difficulty with climbing stairs [17].

The most significant predictors for dissatisfaction include failing to meet expectations, (10.7x greater risk), a low one-year Western Ontario and McMaster University Arthritis Index (WOMAC) (2.5x greater risk), preoperative pain at rest (2.4x greater risk), and a postoperative complication requiring hospital readmission (1.9x greater risk) [18]. Despite the introduction of robot-assisted TKA with the goal of offering more accurate angular measurements, patient satisfaction has not improved. Song et al. reported no difference in Hospital for Special Surgery (HSS) or WOMAC scores between 50 conventional manual TKAs and 50 robotic TKAs at two years of follow-up [19,20]. Liow et al. conducted a prospective randomized trial and found no difference between groups with respect to the Oxford Knee Score (OKS) and Knee Society Score (KSS) at two years of follow-up [21]. Yang conducted a prospective cohort study on 71 robotic TKAs versus 42 conventional jig-based TKAs and found no difference in HSS or WOMAC scores at a minimum of 10 years of follow-up [22]. Cho et al. found no difference in WOMAC, OKS, KSS, or SF-12 at a minimum of 10 years of follow-up between 155 robotic TKAs and 196 conventional jig-based TKAs [23]. While the ability to accurately measure the MA has improved, patient satisfaction and clinical outcomes have not followed.

**Figure 2:**
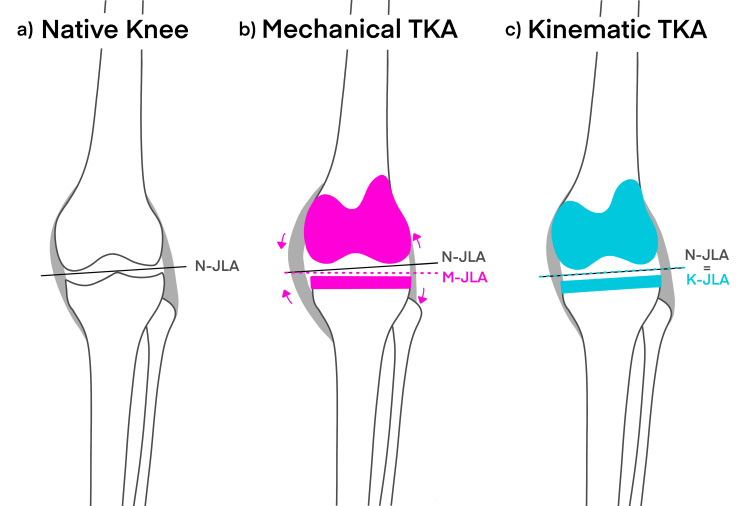
A comparison of knee joint-line angles and soft-tissue tension between a) native knee, b) mechanically aligned total knee arthroplasty, and c) kinematically aligned total knee arthroplasty. The arrows in Figure 2B depict changes in soft-tissue tension resulting from the altered joint-line angle, which in this case leads to decreased tension in the medial soft tissue and increased tension in the lateral soft tissue. TKA=total knee arthroplasty; N-JLA=native knee joint-line angle; M-JLA=mechanically aligned total knee arthroplasty joint-line angle; K-JLA=kinematically aligned total knee arthroplasty joint-line angle.

Some surgeons express hesitancy to perform a kinematically aligned TKA due to its production of a joint line with increased varus relative to the MA of the tibia (Figure 2) [7].

It is thought that aligning the joint with that of the patient’s native anatomy may lead to poor function, early catastrophic failure, and threaten long-term implant survivorship. While there is minimal long-term data on kinematic TKAs, kinematic alignment has not produced the anticipated increased failure rates in short-term studies. As shown in a Level 1 randomized clinical trial, kinematically aligned TKA provided better pain relief, function scores, and postoperative range of motion than mechanically aligned TKA after two years [1,6,24]. This may be attributable to the valgus pull on the joint line while weight bearing, shifting the kinematically aligned varus knee to the neutral alignment that is currently believed to improve TKA mechanics [8]. A prior study by Hutt et al. determined that the joint line perpendicular to the KA is nearly perfectly parallel to the floor, further advocating for a reconsideration of the gold standard for TKA protocols [24]. Matsumoto et al. also found that patients with a kinematically aligned TKA bore weight centrally across the prosthesis during gait, demonstrating improved joint-line parallelism to the floor in comparison to mechanically aligned TKAs [13]. This study further compared patient knee alignment during gait between the KA and MA TKA groups, concluding that kinematically aligned TKA patients demonstrated a more natural gait likely as a result of their increased joint-line parallelism to the floor. These results support studies reporting early and mid-term superior clinical outcomes in KA TKA compared to MA TKA [1,6,24]. The neutralized weight distribution on the polyethylene while weight-bearing may decrease concerns for early implant failure in kinematically aligned TKA, a technique that also minimizes the need for soft-tissue release with a surgical alignment closely matching the patient’s native joint line [11,25].

Given the proposed benefits of improved joint-line parallelism relative to the floor, the aim of our study was to analyze trends of the KA and MA on preoperative radiographs. We attempted to define certain landmarks where the KA and MA intersect. When comparing the MA and KA to the origin point from the center of the femoral head, the KA crossed the knee more laterally at the tibial plafond and often through the distal tibiofibular joint. The average angular difference between MA and KA was 2.957° of valgus (95% CI 2.488-3.427) when measured from the MA, which often led to the tibiofibular joint and the inferior weight-bearing portion of the calcaneus distally. The line from the center of the hip to the inferior calcaneus, or hip-to-calcaneal axis [26], followed a near-identical path to the KA line we had measured. We hypothesize that because the calcaneus is the weight-bearing bone of the lower extremity that transmits body weight to the floor [27], a proper estimation of the optimal knee alignment with regard to parallelism to the floor is the line from the femoral head to the calcaneus, which we found to be in neutral position to the KA. 

Our inclusion criteria were based on radiographic joint space narrowing using the Kellgren and Lawrence system for the classification of osteoarthritis [14,15]. We only selected the subjects with osteoarthritic severity of Grade ≤1, which includes patients with definite absence of x-ray changes of osteoarthritis or subjects with doubtful joint space narrowing and possible osteophytic lipping. One limitation of our study is that the radiographic measurements were only done by one observer. However, measurements were repeated by the observer to ensure intra-observer reliability. While there could be variation in these radiographic measurements if measured by multiple observers, previous studies have found sufficient intra- and inter-observer reliability in human assessment of lower limb alignment on standing long-leg radiographs [28,29]. Another limitation of our study is that the knee joint line in the bipedal stance phase does not account for dynamic loading in the gait cycle. Thus, it cannot be concluded that superior parallelism to the floor in KA versus MA is conserved from the bipedal stance phase throughout the gait cycle [30]. Additionally, further studies are needed to assess patient satisfaction after TKA with respect to joint parallelism to the ground. Despite these limitations, our results show that utilizing kinematic alignment results in a joint that is more parallel to the floor in the stance phase. In addition, due to its similarity to the KA and anatomical significance in weight-bearing distribution, further investigation into the hip-to-calcaneal axis as an approximation of the joint line is warranted.

## Conclusions

Our study found that the kinematic joint line was more parallel to the ground in the bipedal stance phase compared to the MA. These findings challenge the belief that the kinematic TKA technique, often resulting in a slightly varus joint line, may lead to early loosening due to unequal weight distribution across the implant. The increased parallelism to the ground of the KA versus the MA during the bipedal stance phase suggests a more even load distribution across the knee. This neutralized coronal alignment in the stance phase may in part explain why kinematic TKAs have yet to demonstrate the predicted early loosening in early and mid-term studies. Further long-term studies are still needed to determine the long-term survivorship of kinematically aligned TKAs. Investigation into postoperative patient satisfaction scores in relation to joint parallelism to the ground is also warranted. In addition, further studies should investigate the hip-to-calcaneus axis as an approximation of the joint line considering its anatomical significance in weight bearing.
